# Mannitol Enhances the Antinociceptive Effects of Diphenhydramine as an Alternative Local Anesthetic

**DOI:** 10.1155/2020/7934164

**Published:** 2020-11-26

**Authors:** Jo-Young Son, Jae-Seong Lim, Jae-Hyung Park, Jae-Hyeong Park, Myeong-Shin Kim, Jung-Ho Park, Jun-suk Oh, Hyun-Wu Yoon, Jin-Sook Ju, Dong-Kuk Ahn

**Affiliations:** Department of Oral Physiology, School of Dentistry, Kyungpook National University, Daegu, Republic of Korea

## Abstract

Mannitol has recently been reported to be effective in enhancing the antinociceptive efficacy of lidocaine. No single study to date, however, has compared diphenhydramine with and without mannitol for nociceptive processing as an alternative local anesthetic. In this study, we examined the antinociceptive efficacy enhancements of diphenhydramine when combined with mannitol. Male Sprague-Dawley rats weighing 230–260 g were used in a hot plate test to evaluate the antinociceptive effects of diphenhydramine. All chemicals were dissolved in isotonic normal saline and administered subcutaneously into the plantar surface of the right hind paw at 10 min before the hot plate test. A subcutaneous injection of 0.5% or 1% diphenhydramine produced significant inhibition of the withdrawal latency time compared with the vehicle treatment. Antinociceptive effects appeared 10 min after the diphenhydramine injections and persisted for over 30 min. The antinociceptive effects of 1% diphenhydramine were not statistically different from those of 1% lidocaine. Although a subcutaneous injection of a 0.5 M mannitol solution alone did not affect the withdrawal latency time, 1% diphenhydramine with 0.5 M mannitol significantly enhanced antinociception. A subcutaneous injection of 1% diphenhydramine with epinephrine (1 : 100,000) solution did not increase the antinociceptive effect of the diphenhydramine. These results suggest that diphenhydramine with mannitol can be used as an alternative local anesthetic.

## 1. Introduction

It is well known that lidocaine blocks pain by inhibiting the propagation of the action potential caused by the inactivation of voltage-gated Na^+^ channels in the neuronal cell membrane [1]. Lidocaine is, therefore, commonly used as a local anesthetic after administration via infiltration or topical application for peripheral nerve blocks. Notably, however, local anesthetics containing lidocaine produce several unwanted side effects. The high plasma levels of lidocaine typically produce systemic toxicity of local anesthetics because of their high absorption following injection. In addition to lidocaine overdose, frequently reported adverse effects of local anesthetics include vasovagal syncope and epinephrine reactions. Although a low incidence of allergic reactions has been reported in dentistry because of the use of amide-type as opposed to ester-type compounds of local anesthetics [2], a recent meta-analysis has revealed that 19.3% of individual case reports describe adverse drug reactions in the patient's drug allergy history [3].

Antihistamine compounds have been shown to have local anesthetic properties [4], with diphenhydramine being the most effective and least toxic member of this family [5, 6] because diphenhydramine has structural similarities with other neural blocking agents [7]. Hence, antihistamines have been proposed as alternative local anesthetics for canine-allergic patients undergoing pediatric, gastroenterologic, urologic, and anesthesiologic treatments [8–11]. Moreover, antihistamines can be used in dentistry, although only a few studies have demonstrated the efficacy of diphenhydramine as a local anesthetic in dental procedures [2, 6, 7, 12, 13]. Hence, when conventional local anesthetics are contraindicated because of the hypersensitivity of the patient, diphenhydramine is a viable alternative [14].

Recently, the addition of mannitol has been reported to be effective in enhancing the antinociceptive efficacy of lidocaine [15]. To date, however, no single study has compared diphenhydramine with and without mannitol for nociceptive processing as a local anesthetic solution. Thus, we conducted our present animal study to examine the antinociceptive efficacy of diphenhydramine administered via a subcutaneous injection, and whether the efficacy is enhanced by the addition of mannitol.

## 2. Materials and Methods

### 2.1. Animals

We used 84 male Sprague-Dawley rats weighing 230–260 g in the present study. These animals were maintained at a constant temperature of 23 ± 1°C on a 12 : 12-hour light-dark cycle. Food and water were freely available. All of our current procedures involving the use of animals were approved by the Institutional Care and Use Committee of the School of Dentistry, Kyungpook National University (no. 20180108). Our experiments were also conducted in accordance with the ethical guidelines for the investigation of experimental pain in conscious animals of the International Association for the Study of Pain, and blind protocols were used. The sample size was seven animals per group, and each rat was used only once.

### 2.2. Hot Plate Test

A hot plate test was used to evaluate the antinociceptive effects of diphenhydramine via a hot plate analgesia meter (Ugo Basile, Italy). On the testing day, the animals were placed in the observation cage with the hot plate instrument 1 h before the analysis to acclimatize them. The temperature of the hot plate was maintained at 55°C during the experiment. The individual animal was placed on the surface of the plate, and the withdrawal latency time to nociceptive behavior such as withdrawing, jumping, or licking of the right hind paw was measured. The cutoff time of the hot plate test was 30 sec. The withdrawal latency times were measured up to 60 minutes at 10-minute intervals after drug injection.

### 2.3. Chemical Administration

Diphenhydramine, lidocaine, mannitol, and epinephrine were purchased from Sigma-Aldrich (St Louis, MO). All chemicals (50 *μ*L) were dissolved in isotonic normal saline and administered 10 min before the hot plate test. Diphenhydramine (0.5% or 1%) solution was administered subcutaneously into the plantar surface of the right hind paw because intradermal 1% diphenhydramine produced greater anesthesia than placebo and equivalent anesthesia to 1% lidocaine in the previous study [9, 14]. Changes in the withdrawal latency time of the right hind paw were examined after the injection. Additionally, 1% lidocaine was subcutaneously injected to compare its antinociceptive effects with those of diphenhydramine. To investigate whether the antinociceptive efficacy of diphenhydramine had been enhanced, changes in the withdrawal latency time of the right hind paw were also examined after a subcutaneous injection of 1% diphenhydramine in combination with 0.25 M or 0.5 M mannitol solution. Moreover, to examine the additive effects of epinephrine (1 : 100,000), changes in withdrawal latency were measured for 1% diphenhydramine with epinephrine or 1% diphenhydramine with epinephrine and 0.5 M mannitol. A normal saline vehicle was administered as a control.

### 2.4. Statistical Analysis

The behavioral data were analyzed using a repeated-measures analysis of variance (ANOVA), followed by the Holm–Sidak post hoc test. In all statistical comparisons, a *P* value of <0.05 was considered to be significant. All data are presented as the mean ± standard error of the mean (SEM).

## 3. Results

**Figure 1:**
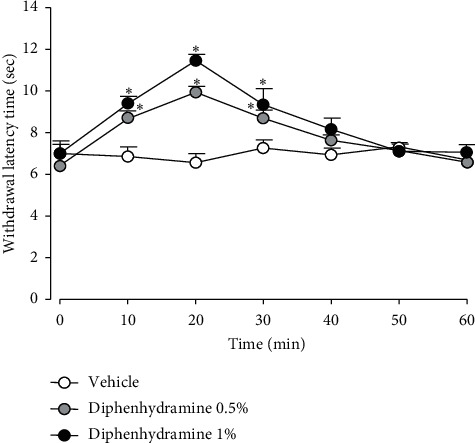
Antinociceptive effects of diphenhydramine on the withdrawal latency time after a subcutaneous injection into the plantar surface of the right hind paw. The injection of 0.5% or 1% diphenhydramine produced a significant inhibition in withdrawal latency compared with the vehicle treatment. The values shown are the mean ± SEM, and there were seven animals in each group. ^*∗*^*P* < 0.05, vehicle vs. diphenhydramine.

Figure 1 illustrates the antinociceptive effects of diphenhydramine on the withdrawal latency time after its subcutaneous injection into the plantar surface of the right hind paw. As the control group, the equivalent administration of the vehicle did not affect the withdrawal latency time whereas both 0.5% or 1% diphenhydramine produced significant inhibition (*F*_(2,15)_ = 3105, *P* < 0.05). These antinociceptive effects appeared 10 min after the injection and persisted for over 30 min and then abated within 50 min.

**Figure 2:**
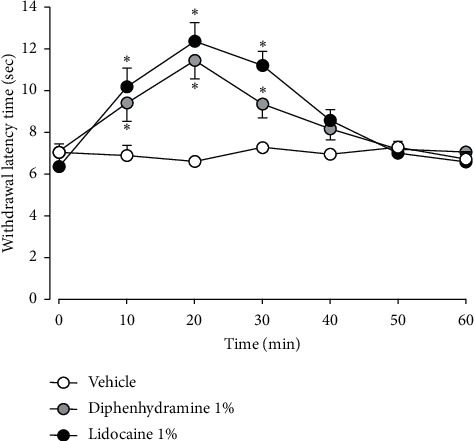
Comparison of the antinociceptive efficacy of 1% diphenhydramine and 1% lidocaine. The subcutaneous injection of either agent significantly inhibited the withdrawal latency time compared with the vehicle treatment, but they were not statistically different from each other in producing this effect. The values shown are the mean ± SEM, and there were seven animals in each group. ^*∗*^*P* < 0.05, vehicle vs. diphenhydramine or lidocaine.

The antinociceptive efficacy of 1% diphenhydramine was compared with the subcutaneous injection of 1% lidocaine, which also significantly inhibited the withdrawal latency time compared with the vehicle treatment (Figure 2; *F*_(2,15)_ = 1335, *P* < 0.05). The appearance and persistence of these antinociceptive effects of lidocaine mirrored those of diphenhydramine and were not statistically different (*F*_(1,10)_ = 763, *P* > 0.05).

**Figure 3:**
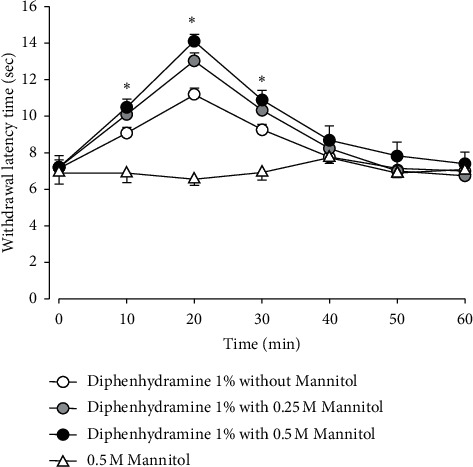
Enhancement of the antinociceptive effects of diphenhydramine by mannitol. As the control group, the subcutaneous injection of 0.5 M mannitol solution alone did not affect the withdrawal latency time. However, the injection of 1% diphenhydramine combined with 0.5 M mannitol significantly enhanced antinociception compared with 1% diphenhydramine alone. The values shown are the mean ± SEM, and there were seven animals in each group. ^*∗*^*P* < 0.05, diphenhydramine with mannitol vs. diphenhydramine without mannitol.

The antinociceptive effects of diphenhydramine were found to be enhanced by the addition of mannitol to the solution, as indicated in Figure 3. A control group injected with the mannitol solution alone did not show any withdrawal latency time effects. The subcutaneous injection of 1% diphenhydramine combined with the 0.25 M mannitol solution increased the antinociceptive effects compared with 1% diphenhydramine alone, but this difference was not significant. However, the combination with 0.5 M mannitol solution significantly enhanced the antinociceptive effects of 1% diphenhydramine (*F*_(2,15)_ = 2364, *P* < 0.05). These antinociceptive effects appeared 10 min after injection and persisted for over 30 min and then these effects abated within 50 min.

**Figure 4:**
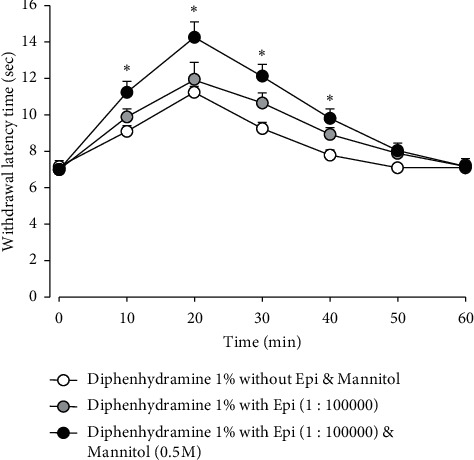
Antinociceptive effects of diphenhydramine combined with epinephrine. The subcutaneous injection of 1% diphenhydramine with epinephrine solution (Epi, 1 : 100,000 dilution) did not increase antinociception compared with 1% diphenhydramine alone. However, the further addition of 0.5 M mannitol to the diphenhydramine and epinephrine solution significantly enhanced the antinociceptive effects. The values shown are the mean ± SEM, and there were seven animals in each group. ^*∗*^*P* < 0.05, diphenhydramine with epinephrine and mannitol vs. diphenhydramine without the epinephrine and mannitol-treated group.

The antinociceptive effects of diphenhydramine combined with epinephrine are illustrated in Figure 4. The subcutaneous injection of 1% diphenhydramine combined with the epinephrine (1 : 100,000) solution did not enhance antinociception (*P* > 0.05), but the inclusion of 0.5 M mannitol to this combination significantly enhanced the antinociceptive effects (*F*_(2, 15)_ = 3942, *P* < 0.05), which again appeared 10 min after the injection and persisted for 40 min. These effects abated within 50 min.

## 4. Discussion

The present study is the first to demonstrate that mannitol enhances the antinociceptive action of diphenhydramine. The subcutaneous injection of diphenhydramine produces significant antinociceptive effects that are comparable to those of lidocaine. Furthermore, the addition of mannitol significantly increases the antinociceptive efficacy of diphenhydramine. These results suggest that diphenhydramine combined with mannitol can be used as an alternative local anesthetic.

Diphenhydramine has been well established as a potent antihistamine within the ethanolamine group and produces anticholinergic, antiemetic, and strong sedative effects. Some clinical studies have demonstrated that diphenhydramine exerts equivalent antinociceptive effects to those of lidocaine in treating minor laceration repair [10, 11]. The use of diphenhydramine has been studied in tooth extraction [5, 14, 16] and inferior alveolar nerve block in human patients [17]. Moreover, the subcutaneous injection of diphenhydramine into the dorsal skin in a prior study in rats produced local analgesia in response to noxious stimuli with an efficacy that was comparable to bupivacaine [18].

Our present animal study has demonstrated that the subcutaneous injection of 1% diphenhydramine into the plantar surface of a rat right hind paw produces significant antinociceptive effects. However, this same treatment in the contralateral shoulder area did not produce any antinociception (data not shown). This suggests that the subcutaneous injection of diphenhydramine at the doses we used in our present analysis did not produce systemic effects. To assess whether the effective antinociceptive doses produced motor dysfunction, rotarod tests were performed after the injection of 1% diphenhydramine. Administration of 1% diphenhydramine produced significant antinociceptive effects, but there was no change in the rotarod test (data not shown). These results imply that the analgesic effect produced by administration of 1% diphenhydramine does not mediated by motor dysfunction or sleepy symptoms. Hence, diphenhydramine can be used as an alternative local anesthetic agent in a clinical setting because its subcutaneous injection produces local anesthesia. In contrast to our present data, the antinociceptive effects of diphenhydramine were not found to be comparable to those of lidocaine in some previous studies [2, 14, 19]. Moreover, this agent has been observed to produce adverse drug reactions including skin necrosis [20] as well as facial edema and extensive nasal swelling as allergic reactions [6]. Taken together, the existing evidence indicates that diphenhydramine should be used cautiously in a clinical setting.

Mannitol is a 6-carbon sugar alcohol used to reduce acutely elevated intracranial pressure after head trauma or to treat kidney failure involving low urine output [21]. Several previous studies have also demonstrated that mannitol significantly enhances the inferior alveolar nerve blocking effects of lidocaine in asymptomatic human patients [15, 22–25]. Our present study, however, is the first to demonstrate that mannitol also significantly augments the local anesthetic efficacy of diphenhydramine. Several previous reports have indicated that the addition of epinephrine attenuates the plasma concentration of lidocaine and thereby minimizes its systemic toxicity and improves the quality and duration of the peripheral nerve block [26, 27]. Notably, we observed that the addition of only epinephrine to diphenhydramine did not increase the local anesthetic efficacy of diphenhydramine. Interestingly however, the further addition of mannitol to the combined diphenhydramine and epinephrine solution significantly increased its antinociceptive efficacy. These antinociceptive effects appeared 10 min after the injection and persisted for over 30 min and then abated within 50 min. These results suggest that mannitol enhances the synergistic antinociceptive efficacy of diphenhydramine as a local anesthetic. Hence, diphenhydramine with mannitol is shown to be a very good substitute for local anesthetics and can be used as an alternative local anesthetic agent in clinical setting. Although we did not evaluate the underlying pathways of this increased antinociception, previous studies have described some of these mechanisms. In brief, hyperosmolar solutions such as mannitol cause the opening of the perineural barrier [28–30], thereby allowing for increased penetrability by macromolecules [28]. Additionally, previous studies in rats have reported that hyperosmolar solutions block or delay the propagation of the action potential in A-type neurons [31]. Further studies are necessary to more precisely elucidate how mannitol exerts its effects on nociception.

The addition of epinephrine to lidocaine is useful in dentistry because it produces less bleeding, less systemic toxicity, and a longer action through its vasoconstriction effects [32]. Moreover, the intrathecal administration of epinephrine has been reported to attenuate the evoked noxious activity of the dynamic range neurons in the spinal cord of cats [33]. In dental procedures, however, the addition of epinephrine to lidocaine increases the risk of adverse drug reactions owing to the different health conditions of the patients and varying tolerance levels to epinephrine. For example, various adverse drug reactions can occur in patients with diabetes or thyroid diseases [34, 35]. In a clinical setting, therefore, doctors often encounter patients who must be anesthetized using an alternative local anesthetic agent. Our current findings demonstrate that mannitol enhances the antinociceptive efficacy of diphenhydramine as a local anesthetic. Hence, when local anesthetics, including lidocaine or epinephrine, are contraindicated because of hypersensitivity or allergic reactions, diphenhydramine combined with mannitol is a possible alternative.

## 5. Conclusions

The subcutaneous injection of diphenhydramine produces significant antinociceptive effects that are comparable to those of lidocaine. This antinociceptive efficacy of diphenhydramine is enhanced by the addition of mannitol to the solution. Diphenhydramine combined with mannitol is a viable alternative local anesthetic.

## Data Availability

The data used to support the findings of this study are included within the article.
